# Adult Intussusception in Hernial Sac: A Case Report

**DOI:** 10.4021/jocmr2009.06.1243

**Published:** 2009-06-21

**Authors:** Imtiaz Wani

**Affiliations:** SS.M.H.S Hospital, Srinagar, India. Email: imtazwani@gmail.com

## Abstract

Adult intussusception is rare. The author is reporting a case of adult intussusception in a 60-year-old female inside of incisional hernia sac, acting as a lead point. The presentation was subacute intestinal obstruction. Ultrasonography was useful adjunct in diagnosis. The site for intussusception was jejunum. Intussusception was seen protruding into hernial sac with adhesions of gut to abdominal wall defect. Resection of involved gut was done.

**Keywords:**

Intussusception; Hernia sac; Jejunum

## Introduction

Adult intussusception is rarely encountered in surgical practices, accounting for 0.003% to 0.02% of all hospital admissions [1], its presentation, etiology and treatment differ from that of children. Adults have an organic lesion within the intussusception in 70% to 90% of cases [2]. Intussusception in adults may represent an underlying malignancy. Diagnosis can be delayed because of its longstanding, intermittent and non-specific symptoms. With more frequent uses of computed tomography in the evaluation of patients with abdominal pain, the condition can be diagnosed more reliably. Most cases are diagnosed at emergency laparotomy and the treatment entails simple bowel resection in most cases.

## Case report

A 60-year-old female presented with painful abdominal swelling along incisional site. There was history of nausea, vomiting and fever. She had undergone laparotomy twelve years back and developed incisional hernia one year later for which she was asymptomatic and did not seek medical advices. General physical examination revealed pulse of 92/minute and blood pressure of 110/80 mm Hg. Systemic examination was normal. On right mid abdomen, abdominal examination revealed 11 × 7 × 1.3 centimeters swelling, which was tender and irreducible with expansile cough, impulse present. Margins of rent were firm and rounded. Percussion note was resonant. Auscultation findings were increased at bowel sounds. Per rectal examination did not reveal anything significant. Biochemistry profile was normal. X- ray of abdomen showed gut distension and a few air fluid levels. Ultrasonography of abdomen showed sausage sign suggesting intussusception. Patient having laparotomy with gut (jejunum) invaginating into a defect of previous abdominal scar was seen. The gut released of adhesions from the hernial sac and the jujenojujenal intussusception mass was seen protruding into sac, where the adhesions acted as a lead point. Resection of intussusception mass was done. Postoperative period was uneventful and is regularly attending our follow up clinics for last 6 months.

## Discussion

Adult intussusception represents 1% of bowel obstructions. The 80-90% of adult intussusceptions have demonstrable organic lesion [3]. About 50% of both enteric and colonic intussusceptions are associated with malignancy. The adult intussusception is classified as per site of intussusception: enteric, ileocolic, ileocecal and colonic. The age of the patient and the anatomic location of the intussusception provide significant input to the etiology, hence the most appropriate surgical procedure desired.in 7.7% of cases are idiopathic adult intussusceptions [4]. Adult intussusception usually has an identifiable pathologic change at the lead point; adhesions, a malignant or benign lesion, endometriosis, drug-related enterocolic lymphocytic phlebitis, mesenteric lymphadenopathy, lymphoid hyperplasia, viral infection, trauma, and pregnancy are considered as predisposing factors [5, 6]. Adults with intussusception present with a myriad of symptoms. The presentation is usually not typical, acute, intermittent, or chronic abdominal condition is found. A number of radiologic investigations have been described in the diagnosis of intussusception. Abdominal ultrasound, computerised tomography scan, barium studies, angiography and radio nucleotide studies are the modalities used. Ultrasound and abdominal computed tomography scan are most ideal in diagnosis of adult intussuscepion showing characteristic “target mass” appearance [7]. Abdomino-pelvic computed tomography (CT) has 78% sensitivity and 100% specificity to diagnose intussusceptions pre-operatively. On CT, changes of intussusception include the early target mass with fascial planes around the mass retained and, in later stages, bowel wall thickening and the characteristic mass with layering effect. Angiographic and radionuclide studies have shown diagnostic efficacy but not used much. Barium studies are confined in diagnosing colonic lesions in adults. Rarely spontaneous resolution of adult intussusception, which has been called “natural cure”, occurs mostly in cases of idiopathic form. Surgical resection of the intussusception is the most often used treatment in adult population. Reduction of the intussusception before resection is controversial, but there is a shift against this, especially in colonic cases [8]. Normally, resection without reduction avoids spillage of contents through accidental perforation during reduction and allows normal bowel to be used for the anastomosis. A formal resection along lymphatic drainage should be performed for all colonic intussusceptions in view of high proportion of these harboring malignant lesions. Surgical treatment can be difficult in gastroduodenal and coloanal intussusceptions, sometimes, it requires innovative techniques. It is possible to employ minimally invasive surgery, especially when the diagnosis is made with confidence preoperatively.
